# Green extraction and phenolic profiling of bitter melon (*Momordica charantia*) using UHPLC-DAD analysis: unveiling its antidiabetic and anticancer potential

**DOI:** 10.3389/fnut.2026.1775934

**Published:** 2026-04-15

**Authors:** Rizwan Ahmad, Aljawharah Alqathama

**Affiliations:** 1 Department of Natural Products, College of Pharmacy, Imam Abdulrahman Bin Faisal University, Dammam, Saudi Arabia; 2 Department of Pharmaceutical Sciences, College of Pharmacy, Umm Al-Qura University, Makkah, Saudi Arabia

**Keywords:** alpha amylase, bitter melon, cytotoxicity, green extraction, phenolic, UHPLC

## Abstract

**Background:**

This study reports for the first time the green extraction and analysis of four phenolics: gallic acid (GA), scopoletin (SC), rosmarinic acid (RA), and resveratrol (RV) in different parts (skin, pulp, seeds) of the fresh and dried fruits of bitter melon (BM). The fruits and their parts were evaluated for comparative anticancer and antidiabetic potential.

**Methodology:**

Ultrasound-assisted extraction (UAE) was used for green extraction, using solvents including ethanol (EtOH), acetone (ACt), ethyl acetate (EtAC), and water (H_2_O), whereas UHPLC-DAD was employed for the simultaneous determination of phenolic content in the three parts of the two different origins of BM fruits (India and Saudi Arabia). The cell lines MRC5, MCF7, and A549 were used for anticancer assays, and *α*-amylase assays were performed for antidiabetic activity.

**Results:**

A high extract yield was observed for the skin part (192.7 mg/1 g; 19.27%) of the dried BM fruit using water as the solvent. The phenolic content yielded higher amounts of (75.04 ppm), RA (45.58 ppm), RV (11.40 ppm), and SC (6.07 ppm) in the dried BM fruit. The cytotoxicity revealed IC_50_ values (μg/mL) of 65.23 ± 1.80 and 65.29 ± 1.77 for I2 (Indian fruit skin) in the MCF7 and A549 cell lines, and 86.74 ± 1.70 for K3 (KSA fruit pulp) in the MRC5 cell line. For *α*-amylase inhibition, the lowest IC_50_ (μg/mL) of 35.89 ± 1.20 was observed for I1 (Indian fruit seeds) samples. Pearson’s correlation, along with the mean differences for the extracts and phenolic content vs. solvents, fresh and dried fruit, and fruit parts, exhibited significant differences (*p* < 0.05).

**Conclusion:**

This study highlights the potential of BM fruit as a rich source of phenolics with notable antidiabetic and anticancer properties. Further *in vivo* studies, such as toxicity assessments against *Artemia salina*, are recommended to elucidate the underlying mechanisms of these bioactivities.

## 1 Introduction

Bitter melon (*Momordica charantia*; BM) is a plant known for its nutritional value and is commonly used in traditional medicine in tropical and subtropical areas of South America, Asia, and Africa (1). The entire plant has been used in various therapeutic systems, with the fruit especially appreciated for its tonic, stomachic, stimulant, emetic, antibilious, and laxative properties (2). In Ayurvedic medicine, BM juice is applied to relieve joint pain, chronic fever, jaundice, and digestive disorders because of its diuretic, laxative, and anthelmintic properties. Topically, it is used to treat skin diseases, burns, and rashes. In African traditional medicine, the fruit, seeds, and leaf juice are used to treat worm infections and inflammation. The leaves are used to address fever and menstrual disorders, while the roots are utilized for syphilis, rheumatism, and various skin conditions. BM is consumed as a dietary remedy for type 2 diabetes in many folk traditions (3). Phytochemical studies of BM have identified over 30 bioactive compounds, making it an important member of the Cucurbitaceae family for both nutritional and medicinal purposes. These substances include heteropolysaccharides, peptides and proteins like momorcharins, saponins, and terpenoids such as cucurbitanes and cucurbitacins, along with flavonoids, volatile oils, sterols, and fatty acids (4). A group of phytochemicals unique to the Cucurbitaceae family, known as cucurbitacins, is abundant in bitter melon (2). These unique phytochemicals, including cucurbitacins, contribute to analgesic, anti-inflammatory, antimicrobial, antiviral, and anticancer effects, while phenolic compounds, including polyphenols and flavonoids, provide antioxidant, antimutagenic, antidiabetic, and anticancer properties. The composition of these bioactive compounds varies depending on the plant part and stage of maturation (5, 6). Extensive research has documented BM’s therapeutic potential. It exhibits antidiabetic activity by improving insulin sensitivity, lowering blood glucose, and reducing complications such as nephropathy and cataract formation (7–9). BM possesses antibacterial, antiviral (including anti-HIV), anthelmintic, antihypertensive, and abortifacient properties. Its anticancer potential has been shown against leukemia, lymphoma, breast cancer, melanoma, and prostate cancer, while antioxidant, anti-inflammatory, and anti-dementia effects have also been reported (10–13). BM lowers blood sugar by preserving and repairing pancreatic *β*-cells, inhibiting enzymes that produce glucose, reducing glucose absorption in the intestines through glucosidase inhibition, and increasing glucose uptake in skeletal muscles. Its anticancer actions involve reactive oxygen species modulation, regulation of cell signaling and cycle progression, induction of apoptosis and autophagy, inhibition of metastasis and angiogenesis, epigenetic modifications, and interactions with DNA, RNA, and proteins leading to cancer immunity and cell death. These diverse mechanisms highlight the multifaceted therapeutic potential of BM (2, 14, 15). These hypoglycemic effects are attributed to various bioactive compounds, including peptides such as polypeptide-p and peroxidase, saponins and terpenoids like momordicoside, and phenolic compounds and flavonoids like kaempferol, quercetin, and isorhamnetin (2, 15, 16).

There is limited information on the phenolic composition of different BM fruit parts using green extraction methods despite extensive studies. This study aims to profile key phenolic compounds—rosmarinic acid, gallic acid, resveratrol, and scopoletin—in fresh and dried BM fruit, including skin, seeds, and pulp, using ultrasound-assisted extraction and UHPLC-DAD analysis. The extracts from BM fruits collected in two geographical regions, Saudi Arabia and India, are evaluated for anticancer and antidiabetic activities. The study examines the relationship between phenolic content and biological activity in these samples. This approach addresses a critical research gap and may provide deeper insights into the therapeutic potential of BM fruit.

## 2 Materials and methods

### 2.1 Sample collection

Samples of bitter melon (BM) fresh fruits from two different geographical origins—India (I) and locally grown in the Kingdom of Saudi Arabia (K)—were obtained from a local market in Al-Khobar, Saudi Arabia. The process of collection, drying, processing, and storage of the samples has been previously described (17). Here, both fresh and dried BM fruits were separated into three distinct segments (seeds, skin, and pulp) before undergoing extraction using the ultrasonic-assisted extraction technique, as detailed below.

### 2.2 Green extraction using ultrasound assisted technique

#### 2.2.1 Method development for green extraction (UAE-MD)

Both fresh and dried BM fruit parts were extracted using UAE (ultrasound-assisted extraction) with green solvents: acetone (ACt), ethanol (EtOH), ethyl acetate (EtAC), and water (H₂O) to extract the desired phenolics—gallic acid (GA), scopoletin (SC), rosmarinic acid (RA), and resveratrol (RS). Specifically, 1 g of each BM fruit part was extracted in 20 mL of the respective solvent using UAE conditions previously reported, with slight modifications to the pulse rate (adjusted from 0.5 to 10 s) (18). A total of 24 samples were prepared, covering three fruit parts (seeds, pulp, skin) in both fresh and dried forms across the three solvents (4 × 3 = 12 for fresh and 4 × 3 = 12 for dried samples). Following extraction, the samples were filtered using Whatman paper, and the solvents were evaporated using a Genevac apparatus. The dried extracts were individually weighed to determine the extraction yield for each BM fruit part in different solvents. Finally, the dried extracts were re-prepared in HPLC-grade EtOH (1 mg/mL) to be analyzed via HPLC-DAD for quantification of the targeted phenolics.

#### 2.2.2 Method validation for green extraction (UAE-MV)

The optimal solvent system (EtOH, ACt, and EtAC) in a ratio of 35:35:30 resulted in higher yield and phenolic content (determined via UHPLC analysis) for the BM fruit parts, which was chosen to validate the green extraction method on a large scale. Herein, UAE was performed using a larger amount of the sample (2 g) and solvent volume (30 mL) from the fruit parts of two different origin BM fruits (Indian and KSA) that were collected. A total of 12 samples from the fresh and dried fruits of the two origins were extracted for skin, seeds, and pulp. All samples were extracted, filtered, dried, weighed for extract yield calculations, and prepared for UHPLC-DAD analysis to simultaneously determine the phenolic compounds (GA, SC, RA, RV), as described previously (Section 2.2.1).

### 2.3 UHPLC-DAD for phenolic profiling (UHPLC-DAD-MDMV)

The analytical method for the simultaneous determination of phenolics (RA, GA, RV, and SC) in the BM fruit samples was accomplished using our previously developed (MD) and validated method (MV) (19). The chromatographic conditions for the analysis of phenolics (GA, SC, RA, and RV) in BM fruit extracts were: isocratic elution for the mobile phase of H_2_O: EtOH (50:50 v/v), a flow rate of 0.9 mL/min, an injection volume of 5 μL, *λ*-max of 253 and 267 nm, and a runtime of 5 min. The phenolic compounds were well separated and showed retention times of 1.35, 2.03, 2.54, and 3.73 min for GA, SC, RA, and RV, respectively. The chromatographic and analytical performance of the method was found to be excellent and reliable, as indicated by the high values of linearity (*r*^2^ = 0.999), accuracy (99–101%), and precision and sensitivity of the method. The validated method has therefore been used in the present work for the analysis of phenolic compounds in the bitter melon extracts for the assessment of biological activity.

### 2.4 Biological activities

#### 2.4.1 Evaluation of selectivity and cytotoxicity

The MTT viability assay was conducted to evaluate the cytotoxicity and selectivity of the 24 extracted samples from UAE-MD for fresh and dried BM fruits and their parts, as previously reported using MCF7, A549, and MRC5 cell lines (20).

Initially, a concentration of 100 μg/mL was tested against the MCF7 cell line. Subsequently, the three tested cell lines were cultured under the same conditions and exposed to the extracts at varying concentrations (500, 250, 100, 50, 10, and 1 μg/mL). After cell treatment at 37 °C in a 5% CO₂ atmosphere for 48 h, MTT was added, and the absorbance of dissolved MTT, which was proportional to the number of viable cells, was measured at 550 nm using a multi-plate reader (BIORAD, PR 4100, Hercules, CA, USA). IC₅₀ values were calculated accordingly.

##### 2.4.1.1 Determination of IC_50_

The second set of experiments for cytotoxicity determination was conducted for those samples showing the highest number of dead cells at 100 μg/mL using MCF7 and A549 cancer cell lines, as well as MRC5 fibroblast cells, using the following range of concentrations (500, 250, 100, 50, 10, and 1 μg/mL). Olaparib was used as a standard anticancer drug in the experiment.

#### 2.4.2 Alpha amylase inhibition

A 500 μg/mL was used as the initial screening concentration of all samples to evaluate the *α*-amylase enzyme activity. Samples that exhibited inhibition were further evaluated at different concentrations (5, 25, 50, 100, 500, 1,000 μg/mL). The inhibitory activity of α-amylase was assessed following the method described by Ahmad et al. (20). A 20 μL aliquot of enzyme reagent was incubated with 20 μL of the sample in each well at 37 °C, followed by the addition of 30 μL of starch solution and an additional incubation for 8 min. This was followed by the addition of hydrochloric acid and the coloring reagent, iodine solution. Absorbance was measured at 550 nm using a multi-plate reader (BIORAD, PR 4100, Hercules, CA, USA). The percentage inhibition of *α*-amylase was calculated as follows:


%inhibition=(A−C/B−C)×100

Where A = reaction mixture absorbance with the sample, B = reaction mixture absorbance without the enzyme, and C = reaction mixture absorbance without the sample.

##### 2.4.2.1 Determination of IC_50_

IC_50_ determination was performed for the samples with the highest inhibition rate for amylase activity. This was conducted by carrying out a second set of experiments using serially diluted concentrations of 1, 10, 50, 100, 250, and 500 μg/mL. Acarbose was used as a standard compound in this experiment.

### 2.5 Statistical analysis

The statistical analysis for the biological activities (%inhibitions and IC50 values) was accomplished using GraphPad Prism (San Diego, CA, USA). The Statistical Package for the Social Sciences (SPSS; V27.0) was utilized for descriptive statistics, correlations, and paired differences studies.

## 3 Results

### 3.1 UHPLC-DAD-MDMV

The detailed data for method development and validation, representing the chromatogram for the simultaneous separation of the phenolic compounds along with the regression values, LOD, LOQ, accuracy, and linearity range, are reported in our previous study (19).

### 3.2 Extract yield

#### 3.2.1 Extract yield for fresh and dried BM fruit

The yield of the seed extract exhibited the highest yield (%) of 28.3 mg/1 g (2.83%) in H_2_O, followed by EtOH (19.6 mg/g, 1.96%), ACt (13.6 mg/g, 1.36%), and EtAC (12.2 mg/g, 1.22%). The skin extract exhibited the highest yield (%) in H_2_O (165.9 mg/g, 1.65%), followed by EtOH (71.1 mg/g, 7.11%), EtAC (55.9 mg/g, 5.59%), and ACt (50.5 mg/g, 5.05%). For the pulp, the extract yield was highest in H_2_O (45.1 mg/g, 4.51%), followed by EtOH (21.2 mg/g, 2.12%), ACt (16 mg/g, 1.6%), and EtAC (13.8 mg/g, 1.38%).

The highest extract yield from the dried fruit of BM and its parts was observed in the skin (192.7 mg/1 g; 19.27%) using H_2_O as the solvent. The extract yield in the fruit parts observed (N = 12) was highest in the skin [H_2_O (192.7 mg/g, 1.92%)], followed by seeds [H_2_O (135.8 mg/g, 1.35%)], and pulp [H_2_O (83.9 mg/g, 8.39%)].

The data for the extract yield in both fresh and dried BM fruit revealed higher yields in water, followed by ethanol, ethyl acetate, and acetone. The fruit part with the highest yield was the skin in both fresh and dried fruit. The data are shown in Table 1.

**Table 1:** Extract yield and phenolic amounts for the fresh and dried BM fruits with descriptive statistics and USE conditions used during method development (MD): 1, 2, and 3 represent seeds, skin, and pulp parts of the fruit; A, E, W, and X represent the green solvents of acetone, ethanol, water, and ethyl acetate, respectively.

S#	Code	Fruit part+ extraction solvent	Sample amount (g)	Solvent volume (mL)	Extract yield (mg)	%yield	USE conditions	Phenolic (ppm)			
								GA	SC	RA	RV
Fresh bitter melon fruit (F)											
1	1AF	Seeds in ACt	1	20	13.6	1.36	Amplitude = 50%; Pulse = 30/10s; Time = 5 min	42.38	–	–	–
2	1EF	Seeds in EtOH			19.6	1.96		2.46	–	–	–
3	1WF	Seeds in H_2_O			28.3	2.83		14.76	–	-	–
4	1XF	Seeds in EtAC			12.2	1.22		7.41	–	4.01	–
5	2AF	Skin in ACt			50.5	5.05		17.99	–	–	–
6	2EF	Skin in EtOH			71.1	7.11		–	–	–	–
7	2WF	Skin in H_2_O			165.9	16.59		15.10	–	–	–
8	2XF	Skin in EtAC			55.9	5.59		–	–	–	–
9	3AF	pulp in ACt			16	1.6		7.32	–	–	–
10	3EF	Pulp in EtOH			21.2	2.12		8.23	–	–	–
11	3WF	Pulp in H_2_O			45.1	4.51		–	–	–	–
12	3XF	Pulp in EtAC			13.8	1.38		5.40	–	–	–
Dried bitter melon fruit (D)											
13	1 AD	Seeds in ACt	1	20	52.3	5.23	Amplitude = 50%; Pulse = 30/10s; Time = 5 min	55.37	6.07	45.58	11.40
14	1ED	Seeds in EtOH			57.6	5.76		75.04	–	19.34	–
15	1WD	Seeds in H_2_O			135.8	13.58		23.44	–	1.93	–
16	1XD	Seeds in EtAC			66.1	6.61		61.67	2.56	32.76	6.60
17	2 AD	Skin in ACt			6	0.6		21.47	1.93	–	–
18	2ED	Skin in EtOH			25.8	2.58		19.27	–	–	–
19	2WD	Skin in H_2_O			192.7	19.27		21.16	–	–	–
20	2XD	Skin in EtAC			6.5	0.65		–	–	3.68	–
21	3 AD	Pulp in ACt			8.4	0.84		53.32	–	17.18	7.28
22	3ED	Pulp in EtOH			31.9	3.19		43.77	–	17.46	–
23	3WD	Pulp in H_2_O			83.9	8.39		20.40	–	0.78	–
24	3XD	Pulp in EtAC			8.8	0.88		43.05	–	4.93	–

Descriptive statistics					
	Minimum	Maximum	Sum	Mean	Std. Deviation
Extract yield	6.0	192.7	1189.0	49.54	50.53
GA	0.0	75.0	559.0	26.62	21.51
SC	1.9	41.9	154.7	17.18	17.73
RA	0.8	104.6	299.1	19.94	26.49
RV	0.0	138.6	257.3	28.59	49.09

#### 3.2.2 Comparison of the yield of fresh and dried fruit extracts

By comparing the extract yield for the tested samples (fresh and dried fruits), data showed that the highest yield observed was for the dried BM fruit (192.7 mg/g) with a %yield of 19.27. The solvent yielding the most was H_2_O for both extracts (fresh and dried fruits), whereas the fruit part resulting in the highest extract yield was the skin for both extracts.

The descending order for the yields in fresh and dried BM fruit is as follows: dried fruit [skin (H_2_O) > seeds (H_2_O) > pulp (H_2_O)] > fresh fruit [skin (H_2_O) > pulp (H_2_O) > seeds (H_2_O)] > the remaining fruit parts and solvents, as presented in Table 1.

### 3.3 Phenolic yield

#### 3.3.1 The phenolic yield for fresh and dried BM fruit

The fresh BM fruit showed a higher amount of GA in the seeds (42.38 ppm) followed by the skin (17.99 ppm). The phenolic RA was detected only in the seeds, whereas SC and RV were not reported in any of the fresh fruit parts.

The phenolic GA (ppm) in the fresh fruit parts was highest in seeds using the solvent ACt (42.38) followed by H_2_O (14.76), EtAC (7.41), and EtOH (2.46). For the skin, more GA was observed in ACt (17.99) followed by EtOH (15.10), whereas for the pulp, more GA was observed in EtOH (8.23) followed by ACt (7.32) and EtAC (5.40). The phenolic RA was observed in the seeds extracted with EtAC only, whereas RA and RV were not detected in any parts of the fresh fruit.

The phenolic content in the dried BM fruit parts revealed more GA (75.04 ppm) followed by RA (45.58 ppm), RV (11.40 ppm), and SC (6.07 ppm).

The dried BM fruit revealed the highest amount of GA (ppm) in the seeds using EtOH (75.02), followed by EtAC (61.67), ACt (55.37), and H_2_O (23.44). The GA in the skin was highest in ACt (21.47) followed by H_2_O (21.16) and EtOH (19.27), whereas for the pulp, the highest yield was observed in ACt (53.32), EtOH (43.77), EtAC (43.05), and H_2_O (20.40).

With regard to RA (ppm) in dried BM fruit, the highest yield was observed in seeds using AC (45.58) followed by EtAC (32.76), EtOH (19.34), and H_2_O (1.93). The skin part showed RV in EtAC (3.68) only, whereas the pulp exhibited a higher amount of RV in EtOH (17.46) followed by ACt (17.18).

SC was observed in the seeds [ACt (6.07 ppm) and EtAC (2.56 ppm)] and the skin part (ACt 1.93 ppm), whereas RV was seen in the seeds [ACt (11.40 ppm) and EtAC (6.60 ppm)] and pulp part (ACt 7.28 ppm).

The phenolic yield in the fresh BM fruit exhibited higher yields for GA and RA in the seeds, whereas RV and SC were below the LOD. For the dried BM fruit, higher phenolic yields for GA, SC, RV, and SC were observed in the seeds. The data for phenolic yield are shown in Table 1.

#### 3.3.2 Fresh BM fruit vs. dried BM fruit phenolic yield

The comparative phenolic yield for fresh vs. dried BM fruit exhibited higher amounts of GA in ACt and EtOH, SC in ACt, RA in EtAC, and RV in ACt for both fresh and dried BM fruits. In addition, the part of the fruit with the highest phenolic yield was the seeds, followed by the skin and pulp.

The comparative analysis of phenolic content in different parts of fresh and dried BM fruits can be summarized as follows: GA, RA, RV, and SC were found in greater amounts in the dried BM fruit compared to the fresh BM fruit (Table 1).

### 3.4 Effect of solvents vs. phenolic amount

#### 3.4.1 Effect on phenolics in fresh BM fruit

##### 3.4.1.1 Individual and total yield/solvent

The phenolic yield on an individual basis in a sample showed higher amounts for GA in 1AF (seeds extracted in acetone) and RA in 1XF (seeds extracted in EtAC). For the yield of phenolics (GA, SC, RA, RV) in a sample/individual solvent, the highest yield (ppm) was observed for 1AF (42.38), followed by 2AF (17.99), 2WF (15.10), 1WF (14.76), and 1XF (11.42). For total phenolic yield (*N = 3*) in the fruit parts/individual solvent, the yield (ppm) ± SD was highest for ACt (67.68 ± 17.97), followed by H_2_O (29.86 ± 8.62), EtAC (16.83 ± 5.71), and EtOH (10.69 ± 4.23).

This concludes that the yield of phenolics on both an individual and total yield basis in various parts of the fresh fruit was highest for ACt, followed by H_2_O, EtAC, and EtOH. For the dried fruit, the yield of phenolics was higher in ACt, followed by EtOH, EtAC, and H_2_O. Table 2 presents the data for the yield of phenolics in various solvents.

**Table 2:** Effect of solvents on the extract yield and phenolic amounts in the fresh and dried BM fruit parts; presenting the individual and total yields/solvent & fruit parts with SD.

BM fruit	Solvents	Sample	Phenolic				Individual yield/solvent	Total yield/ solvent	SD	Individual yield/part					Total yield/ part	Total yield/ fruit	SD
			GA	SC	RA	RV				Part	GA	SC	RA	RV			
Fresh fruit	ACt	1AF	42.38	–	–	–	42.38	67.68	17.97	Seeds	67.00	–	4.01	–	71.02	125.05	26.12
		2AF	17.99	–	–	–	17.99										
		3AF	7.32	–	–	–	7.32										
	EtOH	1EF	2.46	–	–	–	2.46	10.69	4.23								
		2EF	–	–	–	–	0.00			Skin	33.09	–	–	–	33.09		
		3EF	8.23	–	–	–	8.23										
	H_2_O	1WF	14.76	–	–	–	14.76	29.86	8.62								
		2WF	15.10	–	–	–	15.10										
		3WF	–	–	–	–	0.00			Pulp	20.95	–	–	–	20.95		
	EtAC	1XF	7.41	–	4.01	–	11.42	16.83	5.71								
		2XF	–	–	–	–	0.00										
		3XF	5.40	–	–	–	5.40										
Dried fruit	ACt	1 AD	55.37	6.07	45.58	11.40	118.42	219.59	47.68	Seeds	215.53	8.63	99.60	18.00	341.76	617.42	137.14
		2 AD	21.47	1.93	–	–	23.39										
		3 AD	53.32	–	17.18	7.28	77.78										
	EtOH	1ED	75.04	–	19.34	–	94.38	174.88	37.64								
		2ED	19.27	–	–	–	19.27			Skin	61.89	1.93	3.68	–	67.50		
		3ED	43.77	–	17.46	–	61.23										
	H_2_O	1WD	23.44	–	1.93	–	25.37	67.70	2.43								
		2WD	21.16	–	–	–	21.16										
		3WD	20.40	–	0.78	–	21.18			Pulp	160.53	–	40.35	7.28	208.16		
	EtAC	1XD	61.67	2.56	32.76	6.60	103.59	155.25	50.06								
		2XD	–	–	3.68	–	3.68										
		3XD	43.05	–	4.93	–	47.97										

##### 3.4.1.2 Individual and total yield/fruit part

The individual yield of phenolics in the fruit parts showed the highest yield for GA in the seed part (67.0 ppm), followed by the skin (33.09 ppm) and pulp part (20.95 ppm). RA was only detected in the seed part (4.01 ppm).

For the total phenolic yield per individual part, the descending order of occurrence was: seeds (71.02 ppm) > skin (33.09 ppm) > pulp (20.95 ppm), whereas the total phenolic yield for the fruit was 125.05 ± 26.12 ppm. The results indicate that the phenolic yield per fruit part was highest for the seeds, followed by skin and pulp. The data are shown in Table 2.

#### 3.4.2 Effect on phenolics in dried BM fruit

##### 3.4.2.1 Individual and total yield/solvent

The individual phenolic yield in a solvent revealed higher amounts for GA in 1ED (seeds extracted in ethanol) and for RA, RV, and SC in 1 AD (seeds extracted in ACt). For the total phenolic yield (GA, SC, RA, RV) in an individual sample/solvent, the highest yield (ppm) was observed for 1 AD (118.42), followed by 1XD (103.59), 1ED (94.38), 3 AD (77.78), and 3ED (61.23). The total phenolic yield (*N = 3*) in the fruit parts/individual solvent showed a yield (ppm) ± SD for ACt (219.59 ± 47.68), followed by EtOH (174.88 ± 37.64), EtAC (155.25 ± 50.06), and H_2_O (67.70 ± 2.43).

The results for both individual and total yield of phenolics in various parts of the dried fruit showed higher yields for ACt, followed by EtOH, EtAC, and H_2_O, as shown in Table 2.

##### 3.4.2.2 Individual and total yield/fruit part

For the fruit parts, the individual phenolic yield exhibited the highest amount of GA in the seed part (213.53 ppm), followed by the pulp (160.53 ppm) and skin part (61.89 ppm). SC was greater in the seed part (8.63 ppm), followed by the skin part (1.93 ppm), whereas RV was more prevalent in the seeds (18.09 ppm) than in the pulp part (7.28 ppm) of the extracted dried fruit. For RA, the descending order of occurrence was: seeds (99.60) > pulp (40.35 ppm) > skin (3.68 ppm).

The total yield for phenolic compounds per individual part of the fruit was highest for seeds (341.76 ppm), followed by pulp (208.16 ppm) and skin (67.50 ppm). The yield for the total phenolic amount per fruit showed a yield of 617.42 ± 137.14 ppm. The data indicated a higher yield of phenolics per fruit part for seeds, followed by pulp and skin. Detailed data regarding the phenolic yield in solvents and different fruit parts is presented in Table 2.

#### 3.4.3 Comparative analysis for the yield of phenolic in fresh and dried fruit

The yield of phenolics for both extracts (fresh and dried fruits) in various solvents suggested a higher yield for dried fruit in ACt, followed by EtOH and EtAC. The yield of phenolics in the parts of the fruit was highest for the seeds of dried BM fruit, followed by the pulp of dried BM fruit, seeds of fresh BM fruit, skin of dried BM fruit, skin of fresh BM fruit, and pulp of fresh BM fruit.

The total yield for phenolics per fruit indicated a higher phenolic content in dried BM fruit compared to fresh BM fruit, as shown in Table 2.

### 3.5 Cytotoxicity studies

The initial screening for cytotoxicity using the MCF7 cell line revealed the highest inhibition with the lowest % viability (31 ± 0.03) for 2ID (Indian dried BM fruit skin).

The fresh BM fruit showed a higher %inhibition (42 ± 0.03) for 3SF (Saudi BM fruit pulp), followed by 1SF (Saudi BM fruit seeds; 46 ± 0.05). The remaining parts of the fresh BM fruit from Indian and Saudi origins showed an inhibition >50%.

The dried BM fruit from Indian and Saudi origins showed the maximum % inhibition of 31 ± 0.03 for 2ID (Indian dried BM fruit skin), followed by 1SD (36 ± 0.05), 2SD (38 ± 0.03), 3SD (39 ± 0.04), and 3ID (41 ± 0.03). The only sample with inhibition <50% was 1ID (62 ± 0.03). The data regarding the cytotoxicity of the various parts of BM extracts (both fresh and dried fruits) are provided in Table 3.

**Table 3:** Initial screening of the extracts from fresh and dried BM fruit parts for cytotoxicity [MCF7 cell line (MTT 48 h, % of cell viability ± SD)] and amylase activity evaluation [% of enzyme inhibition ± SD].

S#	Origin	Code	Cytotoxicity (MCF7 cell line)	α-amylase inhibition
Fresh BM fruit				
1	Indian seeds fresh	1IF	75 ± 0.05	61 ± 0.05
2	Indian skin fresh	2IF	59 ± 0.05	55 ± 0.04
3	Indian pulp fresh	3IF	58 ± 0.07	65 ± 0.02
4	Saudi seeds fresh	1SF	46 ± 0.05	76 ± 0.03
5	Saudi skin fresh	2SF	69 ± 0.06	77 ± 0.03
6	Saudi pulp fresh	3SF	42 ± 0.03	79 ± 0.06
Dried BM fruit				
7	Indian seeds dry	1ID	62 ± 0.03	83 ± 0.07
8	Indian skin dry	2ID	31 ± 0.03	74 ± 0.04
9	Indian pulp dry	3ID	41 ± 0.03	75 ± 0.06
10	Saudi seeds dry	1SD	36 ± 0.05	68 ± 0.05
11	Saudi skin dry	2SD	38 ± 0.03	80 ± 0.05
12	Saudi pulp dry	3SD	39 ± 0.04	85 ± 0.05

Olaparib and Acarbose were used as positive controls to determine IC₅₀ values.

### 3.6 Alpha amylase activity

The maximum inhibition (85 ± 0.05%) of the *α*-amylase enzyme was noted for 3SD (Saudi dried BM fruit pulp).

The fresh fruit extract exhibited the highest enzyme inhibition (79 ± 0.06%) for 3SF (Saudi fresh BM fruit pulp), followed by 2SF (Saudi fresh BM fruit skin; 77 ± 0.03%) and 1SF (Saudi fresh BM fruit seeds; 76 ± 0.03%). The remaining samples, 1IF, 2IF, and 3IF, showed an inhibition <70%.

The dried BM fruits from Indian and Saudi origins resulted in the highest % inhibition for 3SD (Saudi dried BM fruit pulp; 85 ± 0.05%), followed by 1ID (Indian dried BM fruit seeds; 83 ± 0.07%), 2SD (Saudi dried BM fruit skin; 80 ± 0.05%),3ID (Indian dried BM fruit pulp; 75 ± 0.06%), 2ID (Indian dried BM fruit skin; 74 ± 0.04%), and 1SD (Saudi dried BM fruit seeds; 68 ± 0.05). The detailed data for amylase inhibition are shown in Table 3.

### 3.7 Large-scale application for the developed method

#### 3.7.1 Extract yield for the geographical origins

The fruits from the two varieties (Indian and Saudi) exhibited a higher extract yield (387.18 mg/2 g) for the seeds of the Indian dried fruit (I1), followed by the seeds of the Saudi dried BM fruit K1 (320.8 mg/2 g). The descending order of extract yields for various parts of the fruit from the two varieties showed greater yield for I1, followed by K1, I3, K3, I2, and K2. This suggests a higher yield for Indian origin in the seeds compared to the Saudi origin dried BM fruit: Indian seeds (I1) > Saudi seeds (K1), as shown in Table 4.

**Table 4:** Validation of green extraction with phenolic amounts for dried BM fruit and the biological activities for the fruit parts with IC_50_ values; descriptive statistics for phenolic and biological activities.

S#	Code	Extract yield (mg)	Sample amount (g)	Solvent volume (mL)	USE conditions	Phenolic (ppm)				Cytotoxicity (IC_50_: μg/mL)			α-amylase (IC_50_: μg/mL)
						GA	SC	RA	RV	MCF7	A549	MRC5	
1	I1	387.18	2	30	Amplitude = 40%; Pulse = 40/10s; Time = 10 min	203.66	40.08	20.10	138.57	110.23 ± 1.77	114.05 ± 1.96	121.89 ± 1.86	35.89 ± 1.20
2	I2	245.3				49.69	2.07	6.64	9.04	65.23 ± 1.80	65.29 ± 1.77	98.11 ± 1.76	90.30 ± 1.34
3	I3	309.1				–	36.37	104.64	–	83.04 ± 1.66	115.8 ± 1.90	103.1 ± 1.65	92.56 ± 1.79
4	K1	320.8				99.14	41.89	–	84.42	74.82 ± 1.69	74.46 ± 1.59	96.00 ± 1.34	127.1 ± 1.91
5	K2	210.5				33.78	3.00	12.51	–	76.17 ± 1.03	87.42 ± 0.93	157.6 ± 1.45	159.3 ± 0.92
6	K3	246.9				–	20.71	7.60	–	78.87 ± 1.70	87.51 ± 1.94	86.74 ± 1.70	181.4 ± 1.35
*Olaparib*										2.44 ± 0.59	5.62 ± 0.73	14.39 ± 1.37	
*Acarbose*													78.41 ± 0.67

Descriptive statistics					
DV	Minimum	Maximum	Sum	Mean	SD
GA	0.0	203.7	386.3	64.37	77.52
SC	2.1	41.9	144.1	24.02	18.24
RA	0.0	104.6	151.5	25.24	39.46
RV	0.0	138.6	232.0	38.67	59.05
MCF7	65.2	110.2	488.4	81.39	15.31
A549	65.3	115.8	544.5	90.75	20.52
MRC5	86.7	157.6	663.4	110.57	25.81
α-amylase	35.9	181.4	686.6	114.42	52.70

#### 3.7.2 Phenolic yield for the geographical origin

The phenolic yield for the dried BM fruit from the two origins exhibited higher amounts (ppm) for GA (203.66) in I1 (Indian seeds), SC (41.89) in K1 (Saudi seeds), RA (104.64) in I3 (Indian pulp), and RV (138.57) in I1 (Indian seeds). The total yield (*N = 6*) in the descriptive statistics showed higher amounts (ppm) for GA, followed by RV, RA, and SC. In terms of occurrence in the samples, the highest phenolic amount observed was for SC, followed by RA, GA, and RV.

The descending order for the phenolics in the two different origins of dried BM fruits is suggested: GA was higher in I1, followed by K1, I2, and K2. The phenolic SC was found to be more in K1, followed by I1, I3, K3, K2, and I2, whereas RA was higher in I3, followed by I1, K2, K3, and I2. RV was found more in I1, followed by K1 and I2. Table 4 shows detailed data for the phenolic yield.

This concludes the yield of phenolics by origin and fruit part with a descending order of occurrence: I1 and I3 (GA, RA, and RV) > K1 (GA, RA, and RV) > K1 (SC) > I1 and I3 (SC).

#### 3.7.3 IC_50_ determination for biological activities

##### 3.7.3.1 Cytotoxicity

The IC_50_ values (μg/mL) for the different cell lines showed the lowest values of 65.23 ± 1.80 and 65.29 ± 1.77 for I2 in the MCF7 and A549 cell lines, and 86.74 ± 1.70 for K3 in the MRC5 cell line.

The IC_50_ values (μg/mL) across the three cell lines indicated the lowest values for MCF7 and A549 in the I2 origin, and for MRC5 in the K3 origin. This suggests that I2 and K1 exhibit greater cytotoxic activity in MCF7 and A549 cell lines, whereas K3 shows significant cytotoxic activity in the MRC5 cell line. The data are presented in Table 4.

##### 3.7.3.2 *α*-Amylase activity

For the amylase enzyme, the highest inhibition was observed at the lowest IC_50_ value (μg/mL) of 35.89 ± 1.20 in the I1 sample.

With regard to the different origins of dried BM fruit, the highest inhibition was observed for I1, followed by I2, I3, K1, K2, and K3. This implies that inhibition based on origin and fruit part for amylase may be ordered as: seeds (Indian> Saudi) > skin (Indian> Saudi). The data for amylase inhibition are provided in Table 4.

### 3.8 Statistical analysis

#### 3.8.1 Correlations studies

##### 3.8.1.1 Pearson’s correlation

Bivariate Pearson’s correlation for the data was categorized into DV (phenolic and biological activities) vs. the two different origins, BM fruits and their respective parts. A positive correlation was observed at *p < 0.05* for the following pairs: DV and I2 (0.76; *p* = 0.02), I2 and K1 (0.72; *p* = 0.04), and I2 and K3 (0.79; *p* = 0.01). The pairs of DV and K2 (0.87; *p* = 0.005), DV and K3 (0.88; *p* = 0.004), I2 and K2 (0.95; *p* < 0.001), and K2 and K3 (0.89; *p* = 0.003) exhibited a high positive correlation observed at *p* < 0.01. For I1 and I3, no correlation was noted with any pairs in the dataset. The data for Pearson’s correlation are given in Table 5.

**Table 5:** Bivariate Pearson’s correlation for the dependent variables (DVs), i.e., biological activities and phenolic content vs. the fruit parts of dried BM from Indian and Saudi Arabian origin.

	DV	I1	I2	I3	K1	K2	K3
DV	1	−0.24	0.76*	0.67	0.51	0.87**	0.88**
		0.56	0.02	0.06	0.194	0.005	0.004
I1	−0.24	1	0.21	−0.51	0.46	−0.04	−0.31
	0.56		0.61	0.19	0.24	0.91	0.45
I2	0.76*	0.21	1	0.49	0.72*	0.95**	0.79*
	0.02	0.61		0.2	0.04	<0.001	0.01
I3	0.67	−0.51	0.49	1	−0.15	0.61	0.61
	0.06	0.19	0.2		0.72	0.1	0.1
K1	0.51	0.46	0.72*	−0.15	1	0.65	0.59
	0.19	0.24	0.04	0.72		0.08	0.12
K2	0.87**	−0.04	0.95**	0.61	0.65	1	0.89**
	0.005	0.91	<0.001	0.1	0.08		0.003
K3	0.88**	−0.31	0.79*	0.61	0.59	0.89**	1
	0.004	0.45	0.01	0.1	0.12	0.003	

*Correlation is significant at the 0.05 level (2-tailed), ** Correlation is significant at the 0.01 level (2-tailed).

##### 3.8.1.2 Principal component analysis

**Figure 1:**
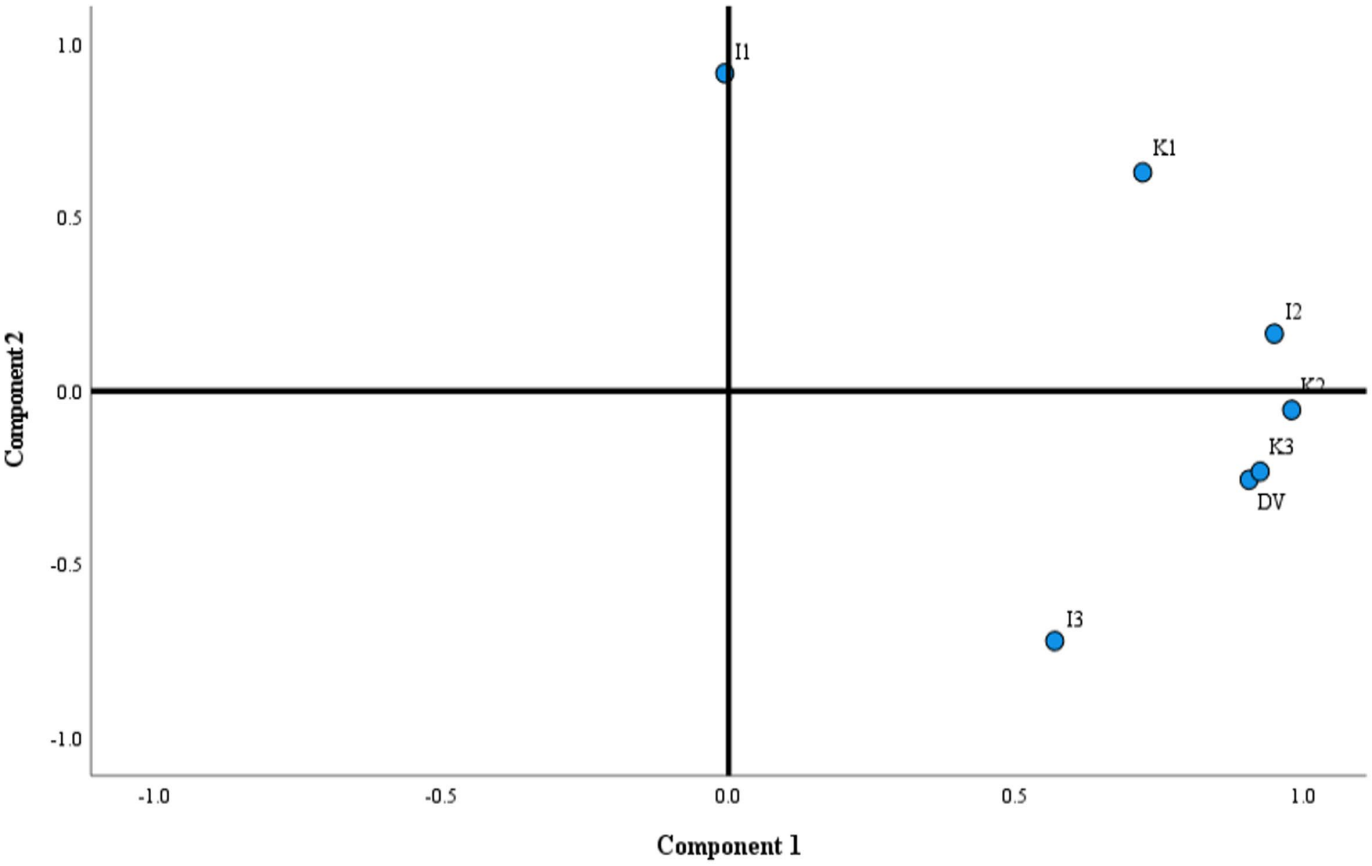
Scree plot loading for the DVs from BM fruit parts (Indian and Saudi origin) in different components of the PCA.

The data were categorized into components based on the % variance using the PCA test. The scree plot (Figure 1) indicated two components with a total variance of 90%, and a single variance of 62.70% for PC1 and 27.30% for PC2. The loading of the data in the two different components (Table 6) showed high % variance and categorization for DV with I2, I3, K2, and K3 in PC1. The components for I1 and K1 were placed in PC2, with the lowest % variability. Thus, this confirms the correlation for DV vs. I2, I3, K2, and K3 as observed in Pearson’s correlation. The KMO and Bartlett’s test of sphericity revealed a high *X2*-value (64.80) with significance <0.001, suggesting the validity of the model.

**Table 6:** PCA with KMO and Bartlett’s test along with loading of the components in PC1 and PC2; paired samples *t*-test with mean, significance, and effect sizes for Cohen’s d and Hedge’s correction.

Principle component analysis (PCA)					
Variables	PC1	PC2	KMO and Bartlett’s test		
DV	0.90	−0.25	Sampling adequacy		0.36
I1	−0.007	0.91	Bartlett’s test of sphericity	Chi. Square	64.80
I2	0.95	0.16		df.	21
I3	0.56	−0.72		Significance	<0.001
K1	0.63	0.72			
K2	0.98	−0.05			
K3	0.92	−0.23			
Individual variance (%)	62.70	27.30			
Cumulative variance (%)	62.70	90.00			

Paired sample *t*-test (paired differences)						
Pairs	Mean	SD	95% CI		*t*	Sig
			Lower	Upper		
I1-DV	93.55	62.81	41.04	146.07	4.21	0.004
I2-DV	43.79	36.41	13.35	74.23	3.40	0.011
I3-DV	62.68	45.69	24.48	100.88	3.88	0.006
K1-DV	70.35	37.34	39.12	101.57	5.32	0.001
K2-DV	61.84	63.06	9.12	114.57	2.77	0.028
K3-DV	53.60	60.85	2.73	104.47	2.49	0.042

Effect sizes for paired samples					
Pairs	Effect	Standardizer	Point Estimate	95% CI	
				Lower	Upper
I1-DV	Cohen’s d	62.81	1.48	0.43	2.49
	Hedges’ correction	66.45	1.40	0.41	2.36
I2-DV	Cohen’s d	36.41	1.20	0.25	2.10
	Hedges’ correction	38.52	1.13	0.23	1.99
I3-DV	Cohen’s d	45.69	1.37	0.36	2.33
	Hedges’ correction	48.33	1.29	0.34	2.20
K1-DV	Cohen’s d	37.34	1.88	0.67	3.05
	Hedges’ correction	39.51	1.78	0.63	2.88
K2-DV	Cohen’s d	63.06	0.98	0.10	1.81
	Hedges’ correction	66.71	0.92	0.09	1.71
K3-DV	Cohen’s d	60.85	0.88	0.03	1.68
	Hedges’ correction	64.37	0.83	0.03	1.59

#### 3.8.2 Mean differences study

##### 3.8.2.1 Mean differences using paired *t*-test

The paired differences exhibited significant differences for the BM fruit parts for the two origins (*p* < 0.05): I1-DV [(*M* = 93.55 ± 62.81), *t* (7) = 4.21, CI = 41.04–146.07], I2-DV [(*M* = 43.79 ± 36.41), *t* (7) = 3.40, CI = 13.35–74.23], I3-DV [(*M* = 62.68 ± 45.69), *t* (7) = 3.88, CI = 24.48–100.88], K1-DV [(*M* = 70.35 ± 37.34), *t* (7) = 5.32, CI = 39.12–101.57], K2-DV [(*M* = 61.84 ± 63.06), *t* (7) = 2.77, CI = 9.12–114.57], and K3-DV [(*M* = 53.60 ± 60.85), *t* (7) = 2.49, CI = 2.73–104.47].

The pairs revealed large sample effect sizes of: 1.48 (Cohen’s d) and 1.40 (Hedges’ correction) for I1-DV, 1.20 (Cohen’s d) and 1.13 (Hedges’ correction) for I2-DV, 1.37 (Cohen’s d) and 1.29 (Hedges’ correction) for I3-DV, 1.88 (Cohen’s d) and 1.78 (Hedges’ correction) for K1-DV, 0.98 (Cohen’s d) and 0.92 (Hedges’ correction) for K2-DV, and 0.88 (Cohen’s d) and 0.83 (Hedges’ correction) for K3-DV. The detailed data regarding paired samples t-test with effect sizes are provided in Table 6.

## 4 Discussion

This study represents the first attempt to develop a green extraction method for phenolic quantification in bitter melon (BM) fruit and its distinct parts (skin, pulp, and seeds). The extraction process was followed by the analysis of phenolics using a previously developed and validated in-house UHPLC-DAD analytical method, which simultaneously determined the presence and concentration of four phenolic compounds: GA (gallic acid), SC (scopoletin), RA (rosmarinic acid), and RV (resveratrol). The developed green extraction and analytical methods were subsequently applied on a large scale to BM fruits from two different geographical origins: Saudi Arabia (K) and India (I). The extracted samples (UAE-MV) were subjected to biological activity assays, including cytotoxicity and *α*-amylase inhibitory activity, to evaluate their potential therapeutic applications. Ultrasonic-assisted extraction was employed as a commonly used method for phytochemical extraction from natural matrices. The process of UAE offers several advantages, including a shorter extraction time, elimination of heating requirements, minimal solvent usage, and higher extraction efficiency. In this study, four green solvents—ethanol (EtOH), acetone (ACt), ethyl acetate (EtAC), and water (H_2_O)—were selected. The fresh BM fruits were separated into three parts (skin, pulp, and seeds), and each part was individually extracted using the selected solvents. The same procedure was applied to dried BM fruit parts. For UAE, previously optimized (18) extraction parameters (50% amplitude, 30/10s pulse, and 5-min extraction time) were employed for all the samples. One gram of each BM fruit part, both fresh and dried, was extracted and filtered. The extraction yield was then calculated prior to phenolic quantification using UHPLC-DAD analysis. A total of 24 samples, including twelve fresh and twelve dried, were analyzed. The highest extraction yield was observed in the dried skin samples using H₂O as the solvent with *N* = 24. A similar trend was observed in fresh BM fruit, where the skin produced the highest yield with H₂O extraction. The results showed clear differences (*p < 0.*05) in extraction yield based on the fruit part, drying status, and solvent type. Overall, H_2_O provided the highest extract yield, followed by EtOH, EtAC, and ACt. This trend in solvent behavior was consistent across the two BM fruit types. However, the fruit parts showed different descending orders for extract yields: skin> pulp> seeds for fresh fruit and skin> seeds> pulp for the dried BM fruit. This is the first report quantifying phenolics in BM fruit, addressing a gap in the literature concerning its individual parts; however, previous studies on natural product extraction have reported higher yields using H_2_O. Budrat’s group extracted phenolics from BM using subcritical water methods, achieving a maximum yield of 52.63 mg gallic acid equivalents per gram of dry weight, a greater amount compared to MeOH extraction (21). Previous studies have demonstrated that water is an effective solvent for extracting plant phenolics. For instance, the highest yield was observed in a water extract from *Micromeria graeca* (47.2%), with a total phenolic content of 360 ± 22.1 mg gallic acid equivalents per gram of dry weight (22). The literature evidence supports the findings of this study.

The phenolic amount was evaluated by analyzing the extracted samples through UHPLC-DAD. The developed method for simultaneous evaluation of phenolics was validated, with a shorter runtime and high accuracy reported previously (18). A notable variation (CI 95%) was observed in the phenolic composition of various parts of the fruit. GA was predominant in the seeds and skin, while RA was mostly found in the pulp. The dried fruit showed a higher phenolic content compared to the fresh fruit. The total phenolic yield varied among the different parts of the BM fruit, with the highest levels found in the seeds, followed by the pith and pulp, and the lowest in the skin. A comparison between fresh and dried samples revealed that the dried BM fruit, especially the seeds and pith, had higher phenolic content than the fresh samples. This reinforces the dried BM fruit and its parts (seeds and skin) as a rich source of phenolics. Studies on the effects of drying temperature have shown that the highest extract yield of BM was obtained when the plant was dried at 40 °C (81.3%), compared to 50 °C, which yielded only up to 73% using the same solvent (23). Additionally, the phenolic composition of BM can vary depending on the drying method used, such as oven-drying or freeze-drying. Significantly higher extract yields were achieved with oven-dried samples compared to freeze-dried ones. The phenolic content in oven-dried plant tissues ranged from 5.39 to 8.94 CAE/g dry material, whereas in freeze-dried samples, it ranged from 4.64 to 8.90 mg (24). In terms of solvent efficiency, acetone yielded the highest amount of phenolic compounds, with ethanol and ethyl acetate following. Water extracted the largest total amount but resulted in the lowest phenolic content. Gallic acid was most abundant in ethanol extracts, while scopoletin and rosmarinic acid were found in higher amounts in acetone extracts. The total phenolic yield of the four compounds was highest in acetone and second highest in ethyl acetate. Considering both individual and total phenolic yields, ethanol, acetone, and ethyl acetate were selected as the optimal solvent system. A solvent mixture of EtOH, ACt, and EtAC in a ratio of 35:35:30 was selected for large-scale phenolic extraction to confirm the efficiency of the developed green ultrasound-assisted extraction method. Previous studies have shown that acetone is an efficient solvent for free phenolic extraction from mulberry leaves, while 70% ethanol is optimal for fenugreek seeds, yielding the highest antioxidant capacity (25, 26). Additionally, EtAC was found to extract the highest phenolic content from *Alocasia longiloba*, followed by MeOH and n-hexane. Furthermore, 25% EtOH was identified as the optimal solvent for extracting phenolics from BM leaves (27). The solvent mixtures employed in previous studies corroborate our data, thereby favoring the selection of an optimal solvent system of EtOH: ACt: EtAC for maximal yield of phenolics (28).

To determine the comparative therapeutic potential of both fresh and dried fruit parts, an initial screening of biological activities was performed. Samples extracted with the optimal solvent system (EtOH: ACt: EtAC) were subjected to cytotoxicity testing on the MCF7 cell line and evaluated for *α*-amylase inhibitory activity. The goal of the initial screening was to identify extracts with over 50% inhibition of the MCF7 cell line and maximal inhibition of α-amylase. Among the twelve tested samples, 2ID (Indian dried BM fruit skin) showed the highest inhibition and the lowest cell viability. The comparative analysis revealed over 50% inhibition for all samples from dried BM fruit, whereas only two samples from fresh BM fruit (1SF and 3SF) exhibited over 50% inhibition. This is likely due to a greater quantity of phenolics (GA, SC, RA, and RV) present in the dried BM fruit. Similarly, the *α*-amylase inhibitory activity ranged from 68–85% for the dried parts and 55–79% for the fresh parts. The comparative analysis categorized the dried fruit and its parts as having dominant cytotoxicity and *α*-amylase activity, making them favorable for further in-depth analysis of the phenolic profile and exploration of their mechanistic roles in biological activities. Recent studies have demonstrated that BM exhibits various pharmacological properties, including cytotoxicity and α-amylase inhibition. Moreover, the whole plant has shown cytotoxic activity against different cell lines, as reported by Fongmoon et al. (29). Their findings indicated LDH release of 51 and 98% at concentrations of 100 and 120 μg/mL, respectively, in HeLa cells, and 70% at a concentration of 180 μg/mL in SiHa cells. Similarly, BM seed extract displayed significant cytotoxicity against human embryonic kidney (HEK293T) cells and human colon tumor (HCT116) cells (30). Additionally, an ethanol fraction of BM demonstrated a potent inhibitory effect on *α*-amylase activity, with an IC_50_ value of 0.27 ± 0.029 mg/mL reported (31). The literature supports our findings on the role of phenolics in biological activities and suggests a preference for dried BM fruit due to its potential therapeutic relevance in cancer and diabetes. Considering the higher extract yield, greater phenolic content, and significant cytotoxic and *α*-amylase inhibitory activities, dried BM fruit was selected for further investigation, including large-scale extraction, phenolic profiling, and determination of IC₅₀ values for cytotoxicity and α-amylase inhibition.

To validate and evaluate the applicability of the developed green extraction and UHPLC-DAD analysis method, the optimal solvent system was tested for large-scale extraction of the different parts of dried BM fruits from two geographical origins: Saudi Arabia (K) and India (I). A larger amount of sample and solvent volume was used to extract phenolics using the aforementioned UAE process. Twelve samples from the three different parts of the two geographical origins of BM fruits (K and I) were filtered, dried, and subjected to UHPLC-DAD analysis for phenolic profiling. Furthermore, the IC_50_ values were determined for the antidiabetic potential using α-amylase inhibition and anticancer potential using three cell lines: MCF7, A549 (lung cancer), and MRC5 (normal fibroblast cell line). The extract yield revealed that I1 (seeds from the Indian dried BM fruit) had the highest amount, followed by K1 (seeds from the Saudi dried BM fruit). The extract yields were observed to be higher for I1, followed by K1, I3, K3, I2, and K2. The phenolic profile indicated higher yields of GA and RV in I1 (seeds from the Indian dried BM fruit), SC in K1 (seeds from the Saudi dried BM fruit), and RA in I3 (pulp from the Indian dried BM fruit). The data suggest higher yields of phenolics in the seed parts of the fruit, with GA being the highest, followed by RV, RA, and SC. In addition, the yield of phenolics in the fruit parts showed a greater amount in the Indian origin BM fruit, in the order of seeds> skin> pulp. Previous phytochemical analysis of BM fruits has revealed an abundance of zinc (2.80 mg/100 g), iron (4.44 mg/100 g), alkaloids, and glycoalkaloids in the seeds, while phenols, flavonoids, and saponins were more prevalent in both the skin and flesh of the fruit (32). To validate and evaluate the applicability of the developed green extraction and UHPLC-DAD analysis method, the optimal solvent system was tested for large-scale extraction of the different parts of dried BM fruits from two geographical origins: Saudi Arabia (K) and India (I). A larger amount of sample and solvent volume was used to extract phenolics using the aforementioned UAE process. Twelve samples from the three different parts of the two geographical origins of BM fruits (K and I) were filtered, dried, and subjected to UHPLC-DAD analysis for phenolic profiling. Furthermore, the IC_50_ values were determined for the antidiabetic potential using *α*-amylase inhibition and anticancer potential using three cell lines: MCF7, A549 (lung cancer), and MRC5 (normal fibroblast cell line). The extract yield revealed that I1 (seeds from the Indian dried BM fruit) had the highest amount, followed by K1 (seeds from the Saudi dried BM fruit). The extract yields were observed to be higher for I1, followed by K1, I3, K3, I2, and K2. The phenolic profile indicated higher yields of GA and RV in I1 (seeds from the Indian dried BM fruit), SC in K1 (seeds from the Saudi dried BM fruit), and RA in I3 (pulp from the Indian dried BM fruit). The data suggest higher yields of phenolics in the seed parts of the fruit, with GA being the highest, followed by RV, RA, and SC. In addition, the yield of phenolics in the fruit parts showed a greater amount in the Indian origin BM fruit, in the order of seeds> skin> pulp. Previous phytochemical analysis of BM fruits has revealed an abundance of zinc (2.80 mg/100 g), iron (4.44 mg/100 g), alkaloids, and glycoalkaloids in the seeds, while phenols, flavonoids, and saponins were more prevalent in both the skin and flesh of the fruit (33). Kampa’s group demonstrated that various phenolic acids, including syringic acid, caffeic acid, and ferulic acid, exhibit antiproliferative activity in the breast cancer T47D cell line. This inhibitory effect was observed as a reduction in the cell percentage in the G2/M phase and an increase in that of the S phase. Additionally, the treatment induced apoptosis by suppressing the levels of the anti-apoptotic protein Bcl-xl while elevating the levels of pro-apoptotic proteins Bak and Fas. These findings highlight the potential of phenolic acids to trigger apoptosis through multiple signaling pathways (34). The results suggest that phenolic compounds contribute to the cytotoxic activity of BM fruit, indicating its potential role in cancer prevention and complementary therapy. Strong *α*-amylase inhibitory activity was observed, especially in sample I1, which consists of seeds from Indian dried BM fruit. This sample showed the lowest IC₅₀ value and contained the highest levels of gallic acid and resveratrol. The dried BM fruit seeds from Saudi origin also exhibited low IC₅₀ values, corresponding to higher contents of gallic acid, scopoletin, and resveratrol. Plant phenolics inhibit *α*-amylase and regulate glucose metabolism by improving *β*-cell function, lowering hepatic glucose production, and enhancing insulin receptor and glucose transporter activity, supporting their role in managing hyperglycemia (35). Furthermore, *in vitro* studies have demonstrated a direct correlation between phenolic content and antioxidant as well as hypoglycemic properties. Higher phenolic composition has been associated with moderate inhibitory activity against *α*-glucosidase and angiotensin-I-converting enzyme, along with strong suppression of α-amylase enzyme activity (36). The strong α-amylase inhibitory activity indicates potential for creating new antidiabetic products. The phenolic composition and related biological activities suggest that BM fruit seeds and skin may be useful sources for therapeutic purposes in cancer and diabetes. This study focuses on evaluating BM fruit quality from different geographical origins based on phytochemical composition. The comparative analysis of fresh and dried BM fruits from Saudi Arabia and India revealed differences in extraction yield, phenolic content, and pharmacological activities. The seeds from the Indian dried fruit showed the highest phenolic levels, cytotoxicity, and *α*-amylase inhibitory activity (37, 38).

Furthermore, several statistical models were applied to evaluate relationships between fruit parts, phenolic content, and biological activities. The Pearson correlation (Table 5) and principal component analysis (Figure 1) showed that samples I1 and I3 had weaker correlations with the other samples and lower variance across components. This suggests that their phytochemical profiles are distinct from the rest. These samples also exhibited the highest individual phenolic levels, with gallic acid and resveratrol being dominant in I1 and rosmarinic acid in I3, while also having the highest total phenolic yield among the twelve samples. This pattern supports the association between higher phenolic content and reduced variability among key components. Sample I1 showed a lower IC₅₀ value for *α*-amylase inhibition compared to the reference drug Acarbose, indicating a clear relationship between phenolic content and biological activity. The paired t-test analysis confirmed differences (CI 95%) among fruit parts from different geographical origins, demonstrating the influence of environmental variation on phytochemical composition and the biological potential of BM fruits.

## 5 Conclusion

This research developed a green extraction method with UHPLC-DAD analysis for the extraction and simultaneous determination of phenolics in various parts of the fresh and dried fruit from two different origins. The findings underscore the significance of BM fruit as an enriched source of phenolics with an established role in cytotoxicity and α-amylase inhibitory activity. The data analysis revealed that the seeds and skin from the dried fruit have potent anticancer and antidiabetic potential. Furthermore, the study serves as a tool to evaluate the effect of geographical variation on phytochemical composition and biological activities. The authors suggest further *in vivo* research studies to provide essential insights regarding the mechanistic role of phenolics in these biological activities and to explore the therapeutic role of BM fruit in various diseases.

## Data Availability

The original contributions presented in the study are included in the article/supplementary material, further inquiries can be directed to the corresponding author.
